# Treatment outcomes in children with Acute lymphoblastic leukemia with versus without coexisting Down's syndrome: A systematic review and meta-analysis

**DOI:** 10.1097/MD.0000000000021015

**Published:** 2020-07-17

**Authors:** Wenjun Liao, Ying Liu

**Affiliations:** a Department of Neonatology; b Department of Oncology, Jingmen No.1 People's Hospital, Jingmen, Hubei, P.R. China.

**Keywords:** acute lymphoblastic leukemia, children, Down syndrome, event-free survival, relapse, treatment-related mortality

## Abstract

**Background:**

Down syndrome (DS) also known as Trisomy 21, is a chromosomal disorder affecting approximately 1 in 732newborns annually in the United States. Children with DS are more likely to develop acute lymphoblastic leukemia (ALL). For the management of pediatric ALL, different treatment protocols have been set up since years. However, ALL children with coexisting DS have shown to have increased therapy-related toxicities compared to those without DS. Therefore, in this study, we aimed to systematically analyze the treatment outcomes in acute ALL children with versus without coexisting DS.

**Methods:**

Electronic databases including the Web of Science, EMBASE, Cochrane Central, MEDLINE, http://www.ClinicalTrials.gov, and Google scholar were searched for publications reporting treatment related outcomes in ALL children with versus without co-existing DS. Several treatment protocols were used accordingly. This study had a long-term follow-up time period ranging from 5 to 10 years. The RevMan 5.3 software was used to carry out this analysis. Odds ratios (OR) with 95% confidence intervals (CI) were used to represent the results post analysis.

**Results:**

A total number of 31,476 children with ALL enrolled between the years 1981 and 2011 were included. Among the total number of children with ALL, 1303 had coexisting DS. Our results showed that event-free survival was similar in ALL children with versus without DS (odds ratio [OR]: 1.34, 95% confidence interval [CI]: 0.51–3.50; *P* = .55). Overall mortality (OR: 1.63, 95% CI: 0.86–3.10; *P* = .13) and participants who achieved clinical remission (OR: 1.04, 95% CI: 0.12–9.29; *P* = .97) were also similarly manifested. However, treatment-related mortality (OR: 4.29, 95% CI: 2.90–6.36; *P* = .00001) and induction failure (OR: 2.77, 95% CI: 1.08–7.07; *P* = .03) were significantly higher in the DS group. Also, total (OR: 1.38, 95% CI: 1.02–1.88; *P* = .04) and bone marrow relapses (OR: 1.29, 95% CI: 1.00–1.67; *P* = .05) were significantly higher in ALL children with DS. Nevertheless, central nervous system relapse (OR: 1.15, 95% CI: 0.60–2.20; *P* = .67), testicular relapse (OR: 0.84, 95% CI: 0.38–1.85; *P* = .87), and other relapses (OR: 1.12, 95% CI: 0.27–4.62; *P* = .88) were not significantly different when these outcomes were separately analyzed.

**Conclusion:**

Based on this analysis of the treatment outcomes in ALL children with versus without DS, event-free survival, overall mortality, and patients who achieved clinical remission were similar during this long-term follow-up time period. However, due to the significantly higher treatment-related mortality, induction failure, and certain relapses in ALL children with DS, new guidelines might have to focus on reconsidering or modifying treatment regimens for ALL children with DS.

## 1 Introduction

Down syndrome (DS) also known as Trisomy 21, is a chromosomal disorder affecting 1 in 732 newborns annually in the United States.^[1]^ Children with DS are more likely to develop acute lymphoblastic leukemia (ALL) compared to children without Trisomy 21.^[2,3]^ During the first 5 years of life, the relative risk for children with DS to develop ALL is >50 times greater than children who do not have DS. Studies have shown the pathophysiology associated with ALL in children with DS to be related to mutation in the hematopoietic transcription factor gene *GATA 1*, a gene that encodes an essential hematopoietic transcription factor.^[4]^ Even, if the prevalence of ALL in children with DS is high, several studies have even shown a 150-fold increase in the incidence of myeloid leukemia.^[5]^ In addition, the estimated incidence of transient myeloproliferative disorder, a pre-leukemia characterized by the excessive growth of immature megakaryoblasts, is approximately seen in 4%–5% of children with DS^[6]^ and about 20%–30% of these children will develop myeloid leukemia by the age of 4 years.^[7]^ Blast cells in myeloid leukemia and transient myeloproliferative disorder also carry acquired mutated genes in the hematopoietic transcription factor GATA1.^[8]^

In the development of ALL in children with DS, several abnormalities in genetic content are involved. For example, the implication of CRLF2, an essential lymphoid signaling receptor, which is dysregulated and overexpressed in >60% of children with DS, has been observed.^[9]^ Another example is the mutation of the Janus Kinase 2 receptors, which could contribute to the development of ALL.^[9]^ Therefore, understanding the mechanisms causing the development of these hematopoietic tumors might be essential to develop medications to prevent progression of the diseases and to predict prognosis.

For the management of pediatric ALL, different treatment protocols have been developed since years.^[10,11]^ Treatment regimens comprised of multiple cytotoxic drugs including doxorubicin, cytarabine, methotrexate, vincristine, and etoposide. However, ALL children with co-existing DS may be more vulnerable to toxic side effects.^[12]^

Therefore, in this study, we aimed to systematically analyze the treatment outcomes in acute ALL children with versus without DS.

## 2 Methods

### 2.1 Search databases and search strategies

The authors searched the Web of Science, EMBASE, Cochrane Central, MEDLINE, http://www.ClinicalTrials.gov, and Google scholar from July to September 2019 for publications reporting treatment-related outcomes in ALL children with versus without co-existing DS using the following Medical Subject Heading (MeSH) terms:

- Acute AND lymphoblastic AND leukemia AND Down's AND syndrome;
- Acute AND lymphoblastic AND leukemia AND Down's AND syndrome AND children;
- Pediatric AND acute AND lymphoblastic AND leukemia AND Down's AND syndrome;
- Leukemia AND Down's AND syndrome AND children;
- Acute AND lymphoblastic AND leukemia AND trisomy 21;
- Acute AND lymphoblastic AND leukemia AND trisomy 21 AND children;
- ALL AND Down's AND syndrome AND children;
- Pediatrics AND ALL AND Down's AND syndrome.

Relevant articles which satisfied the inclusion and exclusion criteria below were then filtered.

### 2.2 Inclusion and exclusion criteria

Criteria for inclusion were studies that reported treatment-related outcomes in ALL children with versus without co-existing DS; were published in English language; consisted of relevant data (dichotomous data) associated with the outcomes which were being assessed with their corresponding number of events occurring in the study and the control groups, respectively.

Criteria for exclusion were studies that were case studies, meta-analyses, and literature reviews; did not compare treatment related outcomes in ALL children with versus without co-existing DS; only consisted of children with DS without any comparison with non-DS (absence of a control group); were published in another language apart from English; consisted of irrelevant data (nondichotomous), which could not be used in this analysis; duplicated studies.

### 2.3 Outcomes

All the outcomes which were reported in the original studies have been listed in Table 1.

**Table 1:**
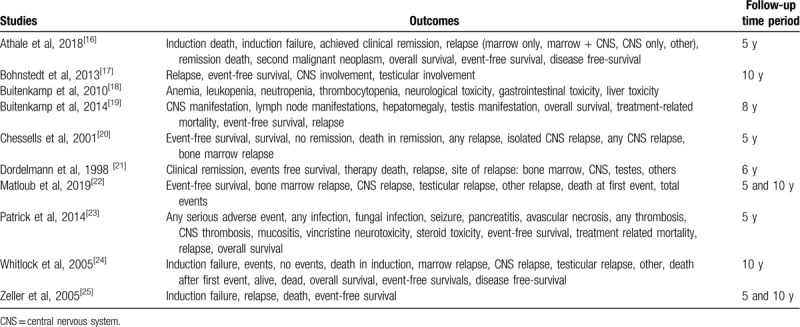
Outcomes and follow-up time periods.

The outcomes which were assessed in this analysis included event-free survival, overall mortality, treatment-related mortality, induction failure, achieved clinical remission, total relapse, central nervous system (CNS) relapse, bone marrow relapse, testicular relapse, and other region relapse.

The mean follow-up time period ranged between 5 and 10 years.

### 2.4 Data extraction and quality assessment

Relevant data were extracted by 2 independent reviewers. First of all, the names, publication year, and data concerning the type of study were retrieved. At a later stage, the total number of participants with and without DS were extracted, followed by the treatments and treatment-related outcomes reported, the total number of events in each category, the follow-up time period, and the baseline features were extracted. Any disagreement during the data extraction or assessment process was resolved by a careful discussion with the most senior, and more experienced doctor, the corresponding author (Y.L.) who was the one to take the final decision.

Furthermore, the methodological features of the studies were assessed using the Newcastle Ottawa Scale (NOS)^[13]^ for observational/retrospective studies and the criteria recommended by the Cochrane Collaboration^[14]^ were used to assess the methodological quality for the randomized trials. Following this assessment, the studies were classified as having a low, moderate, or high risk of bias appropriately.

Ethical approval was not required for this systematic review and meta-analysis.

### 2.5 Statistical analysis

This meta-analysis was carried out by the Cochrane-based RevMan 5.3 software (United Kingdom). Odds ratios (OR) with 95% confidence intervals (CI) were used to represent the results after analysis. A subgroup analysis was considered statistically significant if the corresponding *P* value was ≤0.05. Heterogeneity was assessed by the *I*^2^ statistic test whereby an increasing *I*^2^ value denoted an increased heterogeneity. The statistical model which was used during data analysis was a random-effect statistical model. Sensitivity analysis was also carried out, and publication bias assessment was carried out through visual observation of the funnel plot.

## 3 Results

### 3.1 Search outcomes

A total number of 582 publications were obtained through the search databases (PRISMA guideline) using the respective MeSH terms.^[15]^ A careful assessment of the titles and abstracts (specifically focusing on the key elements of the titles, and the data and results which were reported in the abstracts) was carried out by the authors and based on this assessment, a total number of 539 articles were eliminated since they were not related or linked to the scope or idea of this research topic. Forty-three (43) full-text articles were assessed for eligibility.

**Figure 1:**
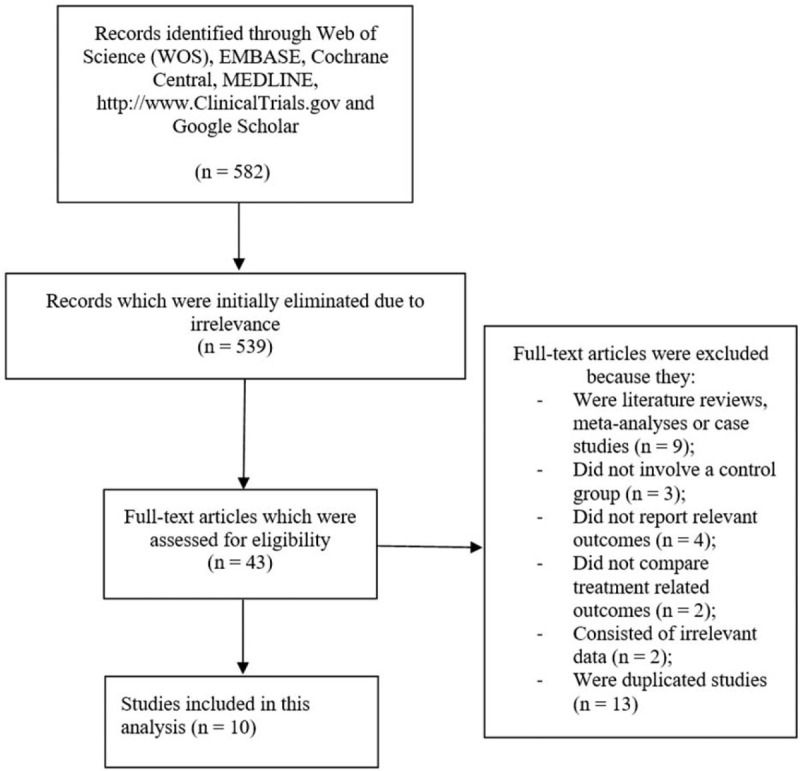
Flow diagram showing the selection of studies to be included in this meta-analysis.

The full-text articles were carefully assessed and further eliminations were carried out based on the inclusion and exclusion features as shown in Figure 1. Finally, only 10 studies^[16–25]^ were confirmed for this analysis.

### 3.2 Main and baseline characteristics

A total number of 31,476 children with ALL enrolled between 1981 and 2011 were included in this analysis whereby 1303 children had DS and 30,173 were non-DS participants. Four studies were trials, whereas 6 studies were observational studies. The general features of the studies have been listed in Table 2. Table 3 lists the baseline features of the children who were involved.

**Table 2:**
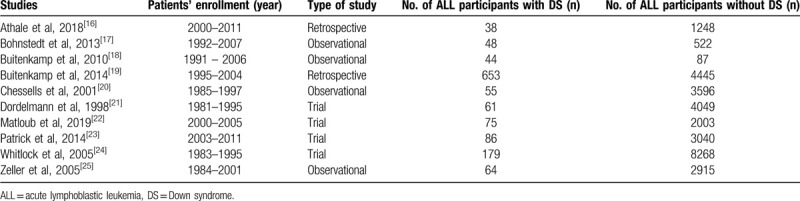
General features of the studies.

**Table 3:**
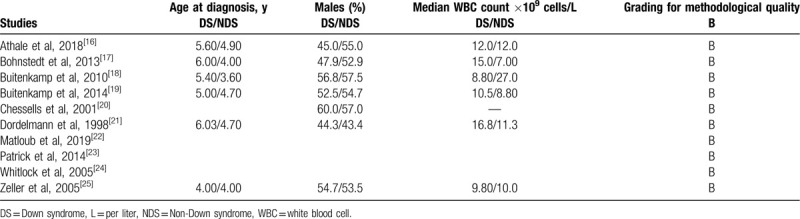
Baseline features.

Based on the methodological quality assessment, an average grade B was allotted representing moderate risk of bias among the trials (assessed by the Cochrane collaboration) and observational cohorts (assessed by the NOS), respectively.

### 3.3 Treatments

Medications were prescribed according to the body weight. Briefly, in Athale et al's study, 2018,^[16]^ ALL children with and without DS were treated based on the Dana-Farber Cancer Institute Acute Lymphoblastic Leukemia Consortium protocols 00-001 (2000–2004) and 05-001 (2005–2011). All the participants received multiagent remission induction consisting of weekly vincristine, prednisolone (40 mg/m^2^/day for a total of 28 days), l-asparaginase, and doxorubicin (total induction dose: 60 mg/m^2^). In protocol 00-001, a single high dose of methotrexate (MTX) (iv 4 g/m^2^) was administered during induction, whereas in protocol 05-001, the participants were administered with a single low-dose MTX (40 mg/m^2^) during induction and then a single high dose of MTX (iv 5 g/m^2^) during the first post induction phase. In Bohnstedt et al's, 2013,^[17]^ and Zeller et al's study, 2005,^[20]^ the participants were treated according to the Nordic Society of Pediatric Hematology and Oncology (NOPHO) ALL92 (1992–2001) or ALL2000 protocol (2003–2007). A 4-week induction therapy was initiated and consisted of vincristine, prednisolone, doxorubicin, and intrathecal MTX, as well as asparaginase. Furthermore, in Buitenkamp et al's study, 2010,^[18]^ and Buitenkamp et al's study, 2014.^[19]^ respectively, treatment was given based on the Dutch Childhood Oncology Group (DCOG) ALL treatment protocol as referenced in detail. In Chessells et al’ study, 2001,^[20]^ the participants were treated on 2 consecutive United Kingdom protocols (MRC UKALL X and XI) briefly consisting of daunorubicin, prednisolone, vincristine, MTX, and l-asparaginase. In Dordelmann et al's study, 1998,^[21]^ the participants were treated based on the ALL Berlin-Frankfurt-Munster Group (BFM) 81, 83, 86, 90 protocols as referenced. Moreover, in Matloub et al's, 2019,^[22]^ and Whitlock et al's study, 2005,^[24]^ the participants were treated according to the Children's Cancer Group (CCG) protocol involving cytarabine, vincristine, dexamethasone, pegaspargase and MTX. At last, the contemporary protocol based on which participants were treated in Patrick et al's study, 2014,^[23]^ has been described previously.

### 3.4 Results of this analysis

**Figure 2:**
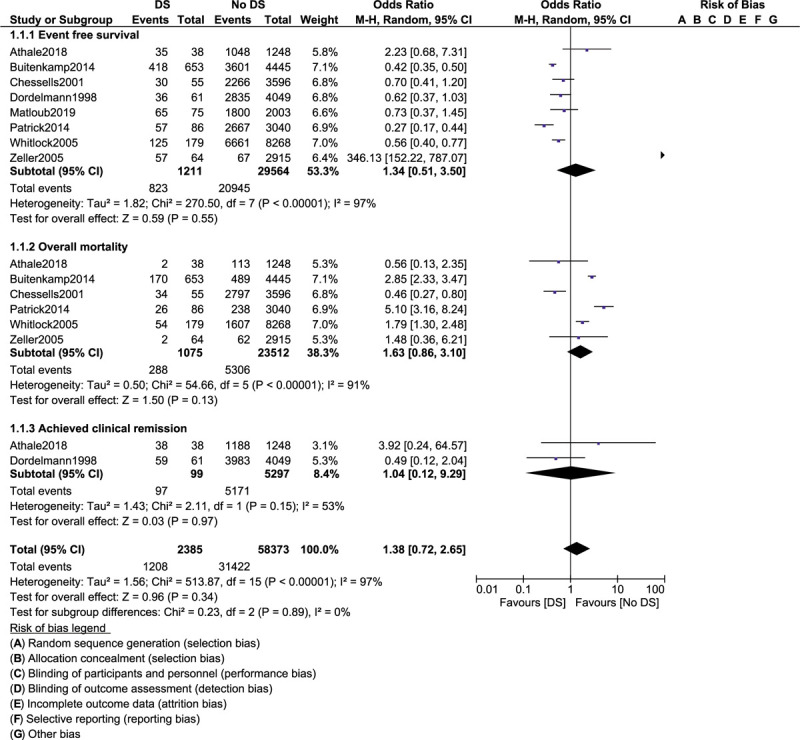
Treatment outcomes in acute lymphoblastic leukemia children with co-existing Down syndrome (part I).

**Figure 3:**
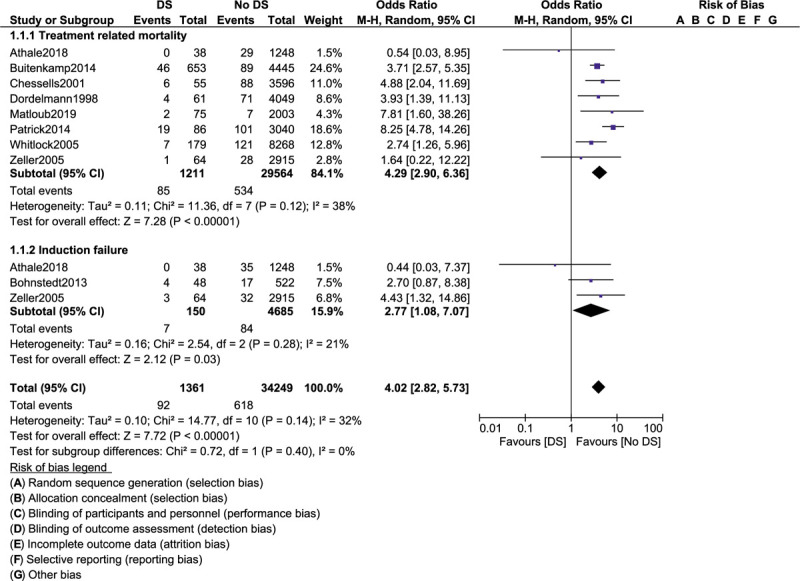
Treatment outcomes in acute lymphoblastic leukemia children with co-existing Down syndrome (part II).

Event-free survival was similar in ALL children with versus without DS (OR: 1.34, 95% CI: 0.51–3.50; *P* = .55) as shown in Figure 2. Overall mortality (OR: 1.63, 95% CI: 0.86–3.10; *P* = .13) and participants who achieved clinical remission (OR: 1.04, 95% CI: 0.12–9.29; *P* = .97) were also similarly manifested (Fig. 2). However, treatment-related mortality (OR: 4.29, 95% CI: 2.90–6.36; *P* = .00001) and induction failure (OR: 2.77, 95% CI: 1.08–7.07; *P* = .03) were significantly higher in ALL children with co-existing DS as shown in Figure 3.

**Figure 4:**
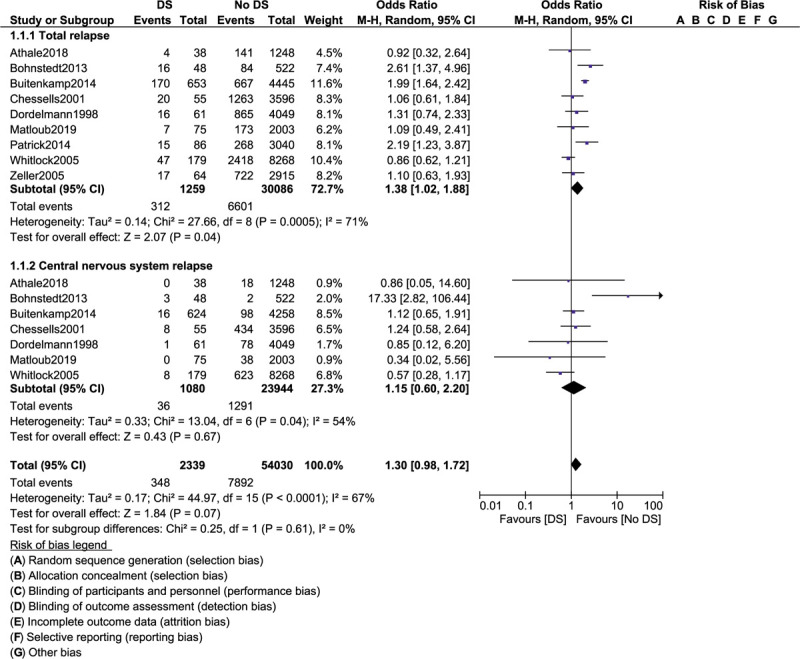
Treatment outcomes in acute lymphoblastic leukemia children with co-existing Dows syndrome (part III).

**Figure 5:**
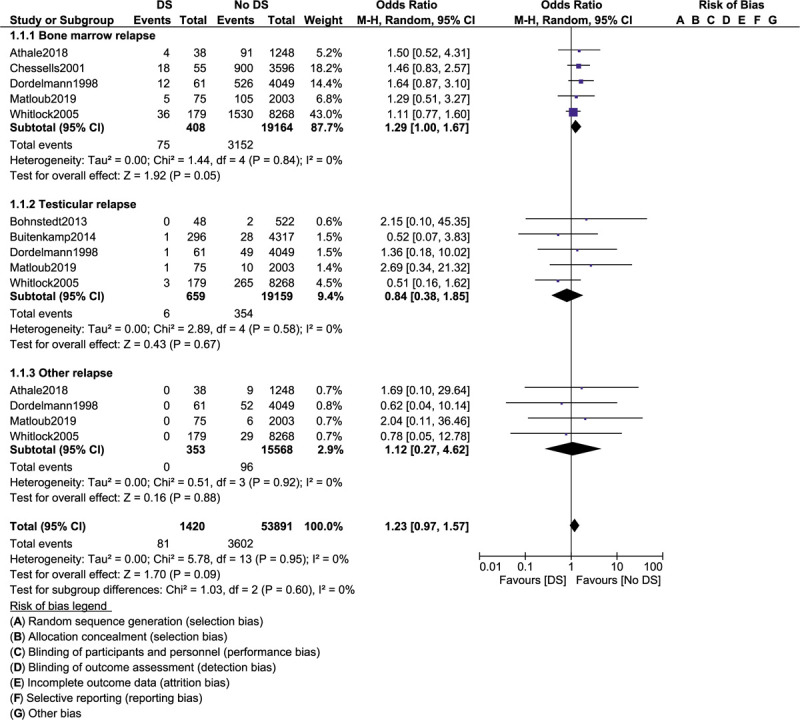
Treatment outcomes in acute lymphoblastic leukemia children with co-existing Down syndrome (part IV).

Total relapse (OR: 1.38, 95% CI: 1.02–1.88; *P* = 0.04) and bone marrow relapse (OR: 1.29, 95% CI: 1.00–1.67; *P* = .05) were also significantly higher with DS as shown in Figures 4 and 5. However, central nervous system relapse (OR: 1.15, 95% CI: 0.60 – 2.20; *P* = .67), testicular relapse (OR: 0.84, 95% CI: 0.38–1.85; *P* = .87) and other relapses (OR: 1.12, 95% CI: 0.27–4.62; *P* = .88) were not significantly different when separately analyzed as shown in Figures 4 and 5. The results have been summarized in Table 4.

**Table 4:**
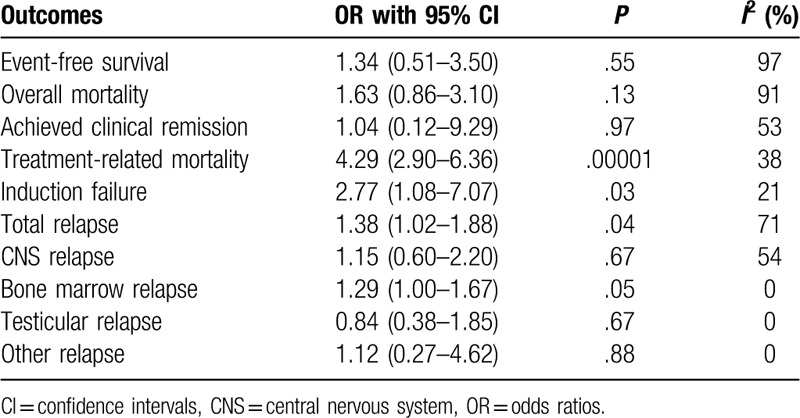
Main results of the analysis.

**Figure 6:**
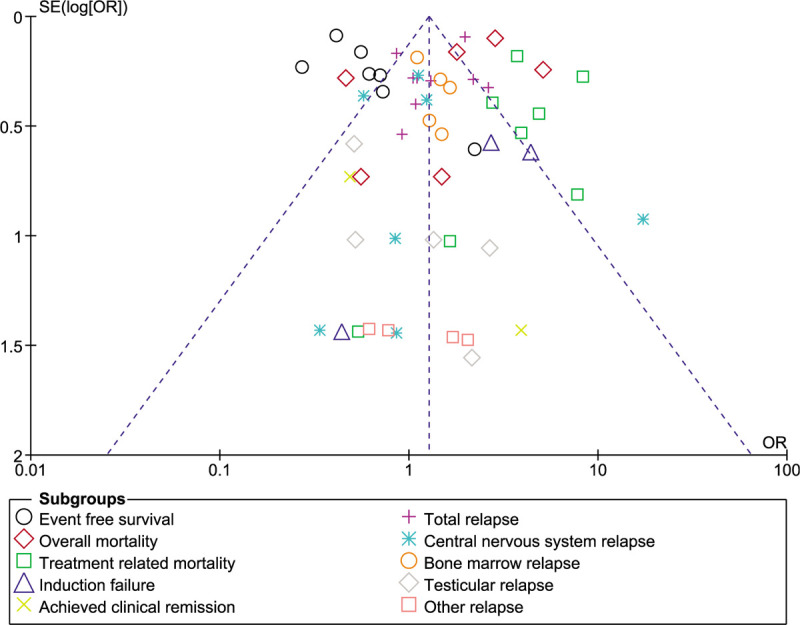
Funnel plot for the visual assessment of publication bias.

Sensitivity analysis showed consistent results throughout. Publication bias was visually assessed by observing the funnel plot represented by Figure 6.

## 4 Discussion

Our analysis showed event-free survival to be similar in ALL children with and without coexisting DS during this long-term follow-up time period. In addition, overall mortality and the number of children achieving remission were also similarly manifested. Even though total and bone marrow relapses were significantly higher in ALL children with co-existing DS, separate analysis did not show any significant difference with CNS, testicular and other relapses compared to ALL children without DS. However, treatment-related mortality and induction failure were significantly increased in the DS group.

A collaborative data analysis on DS children with ALL carried out by the Tokyo Children's Cancer Study Group (TCCSG) and the Kyushu Yamaguchi Children's Cancer Study Group (KYCCSG) showed a 50% 5-year relapse-free period and later, relapse was the main cause of death in these children.^[26]^ In the former study group, the overall survival rate of children was lower among those with co-existing DS.

This current analysis showed an increased risk of treatment-related mortality among pediatric ALL with co-existing DS. To support the results of this analysis, another comparative analysis^[27]^ showed that even though a better treatment response was observed in DS children with ALL, lower event-free survival was due to treatment-related mortality in these children. To further support our results concerning a higher rate of treatment related mortality, data (1982–2004) from the Italian Association of Pediatrics Hematology and Oncology (AIEOP) demonstrated that induction and remission deaths occurred more in ALL children with DS.^[28]^ In addition, featured results from AIEOP showed that leukemia relapse, mainly to the bone marrow, occurred in approximately 31% of the children with DS again supporting this current analysis. The authors further concluded that even though there was a progressive improvement in the DS subgroup with modern therapy, the outcomes were still not as good as those ALL children without DS. Reasons for such a result in ALL children with DS could be related to the biology of the disease, and the respective therapy which could further result in treatment-related toxicities.

Studies showed that there were biological differences between ALL children with versus without DS which could have significant impacts on outcomes and prognosis following treatment. Hyperdiploidy which is referred to >50 chromosomes, has shown to contribute to a better prognosis in children with ALL.^[29]^ However, a significantly lower prevalence of hyperdiploidy was observed in ALL children with DS which might contribute to the poorer post therapeutic outcomes when compared to children without DS.^[24]^ In addition, TEL-AML1 rearrangement is a genetic abnormality which is most frequent in children with ALL. TEL-AML1 rearrangement is normally associated with a good prognosis.^[30]^ However, studies have demonstrated TEL-AML1 re-arrangement to be uncommon in ALL children with DS further contributing to a poor prognosis in these children.^[31]^

Previous studies have shown several mechanisms in children with ALL and DS. A recent study has shown the association of genomic abnormalities of cytokine receptor-like factor 2 (CRLF2) in about 60% of ALL children with DS including CRLF2 translocation with immunoglobulin heavy chain locus at chromosome 14q32, formation of P2RY8-CRLF2 fusion which result in overexpression of CRLF2.^[32]^ Another report from the International BFM Study Group demonstrated that DS confers a rising risk for genetically extreme diverse ALL showing frequent overexpression of CRLF2 associated with mutated Janus kinase 2 (JAK2)^[33]^ which could contribute to unfavorable outcomes and poor prognosis.

In this current analysis, CNS relapse and testicular relapse were similar in children with and without co-existing DS. Another study based on the ALL-BFM treatment regimen showed that dose reduction in the first treatment course decreased severe adverse drug events without increasing the risk of relapse in these children.^[34]^ On the contrary, a nationwide population-based cohort study comparing 5-year leukemia survivors with leukemia-free individuals with DS born in Denmark between 1960 and 2007, and in Sweden between 1973 and 2009, showed that relapse was the major reason for mortality and hospitalization among these children with coexisting DS.^[35]^

When a comparison of the prevalence of favorable and unfavorable biological and clinical characteristics, and adverse drug outcomes was carried out within a total number of 2174 eligible children (ALL with and without DS) enrolled for the CCG-1952 protocol, favorable or unfavorable biological features were less likely among the children with coexisting DS.^[36]^ However, toxicity and hospitalization were more obvious among those with DS compared to the control group. Overall survival was also significantly higher among the ALL patients with coexisting DS.

At last, a 34-year nationwide experience based on the long-term prognosis of children with DS and leukemia, retrospectively from 1968 to 1981, and prospectively from 1982 to 2002, based in Finland, the authors concluded that standard leukemia chemotherapy showed beneficial effects in children with DS.^[37]^ However, due to frequent adverse drug events, the anti-leukemic regimens should better be revised. Also, a matched pair analysis comparing adverse drug events and survival following ALL treatment with an intermediate and a high dose MTX in ALL children with versus without coexisting DS conclusively stated that the treatment which showed efficacy in children with ALL should carefully be incorporated in children with coexisting DS.^[38]^

## 5 Limitations

This analysis has several limitations. First, the total number of ALL children with DS was low compared to the control group. Second, the follow-up time periods for event-free survival and overall mortality varied in several studies (5–10 years). This might have, to a little extent, affected the result of this analysis. Third, there were variations in treatment of the participants with ALL. Different studies used different treatment protocols but which were based on almost similar drugs. However, in our analysis, we were concerned only with the end outcomes, as all the treatments were approved and would result in improvement of the conditions of these children. Fourthly, several subgroup analyses showed a high level of heterogeneity. This was obvious due to the presence of data which were obtained from observational studies and different study designs which would further contribute to the introduction of confounding factors and other types of bias. Only 2 people were involved in the search and extraction of data which could represent another limitation of this study due to potential bias risk. At last, the original studies which have been used in this analysis were not very recent, and did not reflect current therapeutic strategies. However, upcoming studies should be awaited to further investigate this matter.

## 6 Conclusion

Based on this analysis of the treatment outcomes in ALL children with versus without DS, event-free survival, overall mortality, and patients who achieved clinical remission were similar during this long-term follow-up time period. However, due to the significantly higher treatment-related mortality, induction failure, and certain relapses in ALL children with DS, new guidelines might have to focus on reconsidering or modifying treatment regimens for ALL children with DS.

## Author contributions

**Conceptualization:** Wenjun Liao, Ying Liu.

**Data curation:** Wenjun Liao, Ying Liu.

**Formal analysis:** Wenjun Liao, Ying Liu.

**Funding acquisition:** Wenjun Liao, Ying Liu.

**Investigation:** Wenjun Liao, Ying Liu.

**Methodology:** Wenjun Liao, Ying Liu.

**Project administration:** Wenjun Liao, Ying Liu.

**Resources:** Wenjun Liao, Ying Liu.

**Software:** Wenjun Liao, Ying Liu.

**Supervision:** Wenjun Liao, Ying Liu.

**Validation:** Wenjun Liao, Ying Liu.

**Visualization:** Wenjun Liao, Ying Liu.

**Writing – original draft:** Wenjun Liao, Ying Liu.

**Writing – review & editing:** Ying Liu.

## References

[1] Canfield MA Honein MA Yuskiv N National estimates and race/ethnic-specific variation of selected birth defects in the United States, 1999-2001. Birth Defects Res A Clin Mol Teratol 2006;76:747–56. 17051527 10.1002/bdra.20294

[2] Hasle H Clemmensen IH Mikkelsen M Risks of leukaemia and solid tumours in individuals with Down's syndrome. Lancet 2000;355:165–9. 10675114 10.1016/S0140-6736(99)05264-2

[3] Hasle H Clemmensen IH Mikkelsen M [Incidence of cancer in individuals with Down syndrome]. Tidsskr Nor Laegeforen 2000;120:2878–81. 11143409

[4] Harigae H GATA transcription factors and hematological diseases. Tohoku J Exp Med 2006;210:1–9. 16960339 10.1620/tjem.210.1

[5] Lange B The management of neoplastic disorders of haematopoiesis in children with Down's syndrome. Br J Haematol 2000;110:512–24. 10997960 10.1046/j.1365-2141.2000.02027.x

[6] Pine SR Guo Q Yin C Incidence and clinical implications of GATA1 mutations in newborns with Down syndrome. Blood 2007;110:2128–31. 17576817 10.1182/blood-2007-01-069542

[7] Klusmann JH Creutzig U Zimmermann M Treatment and prognostic impact of transient leukemia in neonates with Down syndrome. Blood 2008;111:2991–8. 18182574 10.1182/blood-2007-10-118810 PMC2265448

[8] Wechsler J Greene M McDevitt MA Acquired mutations in GATA1 in the megakaryoblastic leukemia of Down syndrome. Nat Genet 2002;32:148–52. 12172547 10.1038/ng955

[9] Lee P Bhansali R Izraeli S The biology, pathogenesis and clinical aspects of acute lymphoblastic leukemia in children with Down syndrome. Leukemia 2016;30:1816–23. 27285583 10.1038/leu.2016.164 PMC5434972

[10] Yeh TC Liang DC Hou JY Treatment of childhood acute lymphoblastic leukemia with delayed first intrathecal therapy and omission of prophylactic cranial irradiation: Results of the TPOG-ALL-2002 study. Cancer 2018;124:4538–47. 30303520 10.1002/cncr.31758

[11] Sun YN Hu YX Gao L The therapeutic efficacy of pediatric ALL patients with MLL gene rearrangement treated with CCLG-ALL2008 protocol. Eur Rev Med Pharmacol Sci 2018;22:6020–9. 30280786 10.26355/eurrev_201809_15938

[12] Valle M Plon SE Rabin KR Differential in vitro cytotoxicity does not explain increased host toxicities from chemotherapy in Down syndrome acute lymphoblastic leukemia. Leuk Res 2009;33:336–9. 18718659 10.1016/j.leukres.2008.07.011 PMC2637345

[13] Stang A Critical evaluation of the Newcastle-Ottawa scale for the assessment of the quality of nonrandomized studies in meta-analyses. Eur J Epidemiol 2010;25:603–5. 20652370 10.1007/s10654-010-9491-z

[14] Wiley, Higgins JP Assessing risk of bias in included studies, in Cochrane handbook for systematic reviews of interventions. 2008;187–241.

[15] Liberati A Altman DG Tetzlaff J The PRISMA statement for reporting systematic reviews and meta-analyses of studies that evaluate healthcareinterventions: explanation and elaboration. BMJ 2009;339:b2700. 19622552 10.1136/bmj.b2700 PMC2714672

[16] Athale UH Puligandla M Stevenson KE Outcome of children and adolescents with Down syndrome treated on Dana-Farber Cancer Institute Acute Lymphoblastic Leukemia Consortium protocols 00-001 and 05-001. Pediatr Blood Cancer 2018;65:e27256. 29878490 10.1002/pbc.27256

[17] Bohnstedt C Levinsen M Rosthøj S Physicians compliance during maintenance therapy in children with Down syndrome and acutelymphoblastic leukemia. Leukemia 2013;27:866–70. 23138181 10.1038/leu.2012.325

[18] Buitenkamp TD Mathôt RA de Haas V Methotrexate-induced side effects are not due to differences in pharmacokinetics in children with Down syndrome and acute lymphoblastic leukemia. Haematologica 2010;95:1106–13. 20418240 10.3324/haematol.2009.019778 PMC2895034

[19] Buitenkamp TD Izraeli S Zimmermann M Acute lymphoblastic leukemia in children with Down syndrome: a retrospective analysis from the Ponte di Legno study group. Blood 2014;123:70–7. 24222333 10.1182/blood-2013-06-509463 PMC3879907

[20] Chessells JM Harrison G Richards SM Down's syndrome and acute lymphoblastic leukaemia: clinical features and response to treatment. Arch Dis Child 2001;85:321–5. 11567943 10.1136/adc.85.4.321 PMC1718934

[21] Dördelmann M Schrappe M Reiter A Down's syndrome in childhood acute lymphoblastic leukemia: clinical characteristics and treatment outcome in four consecutive BFM trials. Berlin-Frankfurt-Münster Group. Leukemia 1998;12:645–51. 9593260 10.1038/sj.leu.2400989

[22] Matloub Y Rabin KR Ji L Excellent long-term survival of children with Down syndrome and standard-risk ALL: a report from the Children's Oncology Group. Blood Adv 2019;3:1647–56. 31160295 10.1182/bloodadvances.2019032094 PMC6560340

[23] Patrick K Wade R Goulden N Outcome of Down syndrome associated acute lymphoblastic leukaemia treated on a contemporary protocol. Br J Haematol 2014;165:552–5. 24428704 10.1111/bjh.12739

[24] Whitlock JA Sather HN Gaynon P Clinical characteristics and outcome of children with Down syndrome and acute lymphoblastic leukemia: a Children's Cancer Group study. Blood 2005;106:4043–9. 16109782 10.1182/blood-2003-10-3446

[25] Zeller B Gustafsson G Forestier E Nordic Society of Paediatric Haematology and Oncology (NOPHO). Acute leukaemia in children with Down syndrome: a population-based Nordic study. Br J Haematol 2005;128:797–804. 15755283 10.1111/j.1365-2141.2005.05398.x

[26] Goto H Inukai T Inoue H Acute lymphoblastic leukemia and Down syndrome: the collaborative study of the Tokyo Children's Cancer Study Group and the Kyushu Yamaguchi Children's Cancer Study Group. Int J Hematol 2011;93:192–8. 21286878 10.1007/s12185-011-0765-3

[27] Pennella CL Rossi JG Baialardo EM Acute lymphoblastic leukemia in children with Down syndrome: comparative analysis versus patients without Down syndrome. Arch Argent Pediatr 2018;116:e500–7. 30016023 10.5546/aap.2018.eng.e500

[28] Arico M Ziino O Valsecchi MG Italian Association of Pediatric Hematology and Oncology (AIEOP). Acute lymphoblastic leukemia and Down syndrome: presenting features and treatment outcome in the experience of the Italian Association of Pediatric Hematology and Oncology (AIEOP). Cancer 2008;113:515–21. 18521927 10.1002/cncr.23587

[29] Pui CH Raimondi SC Dodge RK Prognostic importance of structural chromosomal abnormalities in children with hyperdiploid (greater than 50 chromosomes) acute lymphoblastic leukemia. Blood 1989;73:1963–7. 2713510

[30] Shurtleff SA Buijs A Behm FG TEL/AML1 fusion resulting from a cryptic t(12;21) is the most common genetic lesion in pediatric ALL and defines a subgroup of patients with an excellent prognosis. Leukemia 1995;9:1985–9. 8609706

[31] Lanza C Volpe G Basso G The common TEL/AML1 rearrangement does not represent a frequent event in acute lymphoblastic leukaemia occuring in children with Down syndrome. Leukemia 1997;11:820–1. 9177434 10.1038/sj.leu.2400651

[32] Buitenkamp TD Pieters R Gallimore NE Outcome in children with Down's syndrome and acute lymphoblastic leukemia: role of IKZF1 deletions and CRLF2 aberrations. Leukemia 2012;26:2204–11. 22441210 10.1038/leu.2012.84

[33] Hertzberg L Vendramini E Ganmore I Down syndrome acute lymphoblastic leukemia, a highly heterogeneous disease in which aberrant expression of CRLF2 is associated with mutated JAK2: a report from the International BFM Study Group. Blood 2010;115:1006–17. 19965641 10.1182/blood-2009-08-235408

[34] Kroll M Kaupat-Bleckmann K Möricke A Methotrexate-associated toxicity in children with Down syndrome and acute lymphoblasticleukemia during consolidation therapy with high dose methotrexate according to ALL-BFM treatment regimen. Haematologica 2020;105:1013–20. 31371414 10.3324/haematol.2019.224774 PMC7109740

[35] Vonasek J Asdahl P Heyman M Late mortality and morbidity among long-term leukemia survivors with Down syndrome: a nationwide population-based cohort study. Pediatr Blood Cancer 2018;65:e27249. 29797653 10.1002/pbc.27249

[36] Bassal M La MK Whitlock JA Lymphoblast biology and outcome among children with Down syndrome and ALL treated on CCG-1952. Pediatr Blood Cancer 2005;44:21–8. 15368546 10.1002/pbc.20193

[37] Rajantie J Siimes MA Long-term prognosis of children with Down's syndrome and leukaemia: a 34-year nation-wide experience. J Intellect Disabil Res 2003;47 (pt 8):617–21. 14641809 10.1046/j.1365-2788.2003.00477.x

[38] Shah N Al-Ahmari A Al-Yamani A Outcome and toxicity of chemotherapy for acute lymphoblastic leukemia in children with Down syndrome. Pediatr Blood Cancer 2009;52:14–9. 18802938 10.1002/pbc.21737

